# Leading by Example: IJIC’s Journey to Strengthen Lived Experience in Research, Improvement, and Scientific Publishing

**DOI:** 10.5334/ijic.8622

**Published:** 2024-03-22

**Authors:** Robin Miller, Viktoria Stein, Heather Penwarden, Eskil Degsell, Ghislaine Rouly, Ivette Fullerton, Susan Royer

**Affiliations:** 1 Health & Social Care, University of Birmingham, United Kingdom; 2 Population Health Management, Leiden University Medical Centre, the Netherlands; 3 Dementia Friendly Honiton & Public Governor Royal Devon NHS Foundation Trust, United Kingdom; 4 Swedish Brain Tumor Assocation and NOCRiiC (Neuro Oncology Clinical Research, innovation, implementation and Collaboration) at Karolinska University Hospital and Karolinska Institute, Sweden; 5 Canada Research Chair in Partnership with Patients and Communities, Canada; 6 The Cihuapactli Collective, United States of America; 7 International Journal of Integrated Care, United Kingdom

**Keywords:** co-production, lived experience, integrated care research, patient involvement, citizen science

Putting people with lived experience of accessing health and/or social care and their families at the heart of integrated care has been the focus of several editorials within IJIC over recent years [1, 2, 3]. This reflects the increasing centrality within wider policy and practice of co-production at both micro (i.e. in the relationships between people and professions) and macro (i.e. in strategic visioning, planning and resource allocation) levels of an integrated care system. Despite the importance of understanding and acting upon the interests and aspirations of people with lived experience and their families, and indeed geographic and other communities, there has been relatively little recognition of this within the research and practice innovations that underpin the articles published within IJIC. The recent review of articles published in IJIC from 2012 to 2022 identified only 14 out of the 560 articles explicitly outlined the approach to involvement in their work [4]. This may reflect that people and families are not included in the design and implementation of these activities or that such engagement is happening, but it has not been detailed within the content provided.

The Editors in Chief and Editorial Board of IJIC have been concerned about this disparity for some time. In part, this is because it does not reflect the values which underpin integrated care and the International Foundation for Integrated Care. But also because we believe that good science should be based around what matters to individuals and communties and this necessitates the generation of evidence together with people with lived experience. An international journal has an important role in supporting academic and practice colleagues to develop their research and improvement skills. By not encouraging authors to share how they sought to embed lived experience within their projects we are therefore missing an opportunity to generate wider learning. Finally, the important contribution of citizen science to generating evidence is receiving justified recognition and again this is not currently reflected in IJIC.

To address these gaps, we (people with lived experience and journal representatives) have been collaborating over a twelve-month period to identify opportunities to strengthen related aspects of IJIC. Working group members were drawn from North America and Europe and had experience of participating and/or leading health and social care research and improvement projects. Our group met four times and considered the overall aims of the journal, review processes, support and development of authors and reviewers, and overall governance of the journal. To inform the discussions, we completed desk top research looking at the practice of other journals and met with representatives from publications who have already made substantial progress in this aspect of their work. The proposals were then shared and endorsed by IFIC and the Editorial Board, resulting in an ambitious plan for how we will embed lived experience within the journal.

The first development will be that from April 2024 IJIC will be introducing the requirement that all submissions to the journal must detail how people with lived experience of health and/or social care and/or families were involved in the development and delivery of the activities which underpin the submission. In research, this could incorporate, how the topic and questions were set, the design of the methodology, the gathering and analysis of data, and identifying implications for policy and practice. In improvement, this could include establishing the need and opportunities, creating and implementing an intervention, and evaluating its impact and process. There will not be a minimal level of involvement mandated as we realise that an element of co-production is not always achievable within the scope, timing or resources but would expect this (and the potential limitations) to be detailed. The guidance for authors and reviewers will be adapted to recognise these new requirements.

The second development will be to recruit people with lived experience on to the IJIC editorial board. As the body which supports the journal in developing its strategy and editorial priorities, it is essential for the board to include a greater diversity of perspectives to provide informed challenge. The new board members will be recruited through an open selection process and (in line with other members) will serve for an initial period of four years. We would hope to attract those from different countries and interests within integrated care. The journal will offer a recognition payment to reflect their contribution if they are not employed in a role through which they can undertake such work. The call for applications will be launched at ICIC24 and then posted on the journal website and distributed throughout our networks.

The third major development will be to recruit a pool of reviewers with lived experience who can contribute to this core aspect of the journal’s processes. In line with other reviewers, these colleagues will consider articles in line with their own interests and expertise. Recognising that this will be a new undertaking for many, we will provide training and support, including the setting up a community of practice in which these reviewers can share learning and challenges. These activities will require additional journal management capacity which will be provided by IFIC as part of wider developments relating to co-production. We plan to begin this process by October 2024.

Once these three building blocks are in place, IJIC plans to further strengthen our engagement with lived experience through developing an article type based around citizen science. We will introduce a process through which the ‘degree’ of influence and involvement within the underlying research and/or improvement can be captured. This will help the journal to understand progress over time and inform a yearly review of lived experience which will be undertaken by the editorial board. The working group which has informed these developments has highlighted once again the value of open, honest, and respectful dialogue and debate. We look forward to future collaboration to help identify further opportunities to embed lived experience within IJIC and to grow our collective understanding.
